# Annual acknowledgement of reviewers

**DOI:** 10.1186/1755-8794-6-6

**Published:** 2013-03-07

**Authors:** Tim Sands

**Affiliations:** 1 BioMed Central, Floor 6, 236 Gray's Inn Road, London WC1X 8HB, UK

## Abstract

**Contributing reviewers:**

The editors of BMC Medical Genomics would like to thank all our reviewers who have contributed to the journal in Volume 5 (2012).

## 

Kotb Abdelmohsen

USA

Ivan Adzhubey

USA

Sher Ali

India

Reid Alisch

USA

Heike Allgayer

Germany

Mark Alter

USA

Jerome Ambroise

Belgium

Marcella Attimonelli

Italy

Charles Auffray

France

Tsukasa Baba

Japan

Manny Bacolod

USA

Liviu Badea

Romania

Matthew Barnett

New Zealand

Jennifer Beane

USA

Erica Bell

USA

Brian Bennett

USA

Douglas Bittel

USA

Esther Black

USA

Hélène Blons

France

Nikola Bowden

Australia

Esteban Braggio

USA

Marcus Breese

USA

Jerome Brody

USA

Murray Cairns

Australia

Jordi Camps

USA

Samuel Canizales

Mexico

Vincent Carey

USA

Luis Carvajal Carmona

UK

Theresa Casey

USA

Peter Celec

Slovakia

Peh Yean Cheah

Singapore

Guang-wu Chen

Taiwan

Yangchao Chen

Hong Kong

Samuel Chong

Singapore

Shimul Chowdhury

USA

Robert Cooper

UK

Maria Lúcia Corrêa-Giannella

Brazil

Richard Cotton

Australia

Josef Davidsson

Sweden

Hao Deng

USA

Robert Dinser

Germany

Christine Disteche

USA

Mikhail Dozmorov

USA

Christine Duarte

USA

John Elefteriades

USA

Theodoros Eleftheriadis

Greece

Serenella Eppenberger-castori

Switzerland

Jing-yuan Fang

China

Harriet Feilotter

Canada

Annika Fendler

Germany

Gang Feng

USA

Gabriella Ferrandina

Italy

Jorge Ferrer

Spain

Jacob Finkelstein

USA

Diego Forero

Colombia

Howard Fox

USA

Maria Fuciarelli

Italy

Terry Furey

USA

Dongliang Ge

USA

Juan Gonzalez

Spain

Marianne Grant

USA

Hank Greely

USA

Anita Grigoriadis

UK

Sergio Grimbs

Germany

Edna Grünblatt

Switzerland

Mireia Guerau-de-Arellano

USA

Dominique Guerrot

France

Balázs Győrffy

Hungary

Benjamin Haibe-Kains

Belgium

Jennifer Harris

Norway

Christina Haston

Canada

Ingrid Hedenfalk

Sweden

Åslaug Helland

Norway

Khalil Helou

Sweden

Ken Hess

USA

Martin Hofmann-Apitius

Germany

Stuart Hogarth

UK

Joanna Holbrook

Singapore

Michael Holick

USA

Mette Hollensted

Denmark

Zihua Hu

USA

Hsuan-Cheng Huang

Taiwan

Xudong Huang

USA

Robert Hudson

USA

Thomas Hughes

UK

Federico Innocenti

USA

Ivan Iossifov

USA

Peilin Jia

USA

Hui Jiang

USA

Glen Jickling

USA

Markus Joerger

Switzerland

Julie Johnson

Australia

Daniel Jordan

USA

Wenjun Ju

USA

Vaijayanti Kale

India

Dongwan Kang

USA

Thomas Karn

Germany

Masaru Katoh

Japan

Seong-Jin Kim

South Korea

Thomas Knudsen

USA

Sakari Knuutila

Finland

Shuhei Komatsu

Japan

Scott Kopetz

USA

György Kosztolányi

Hungary

Urho Kujala

Finland

Naohiro Kurotaki

Japan

Bonnie Lafleur

USA

Leo Lahti

Finland

Xun Lan

USA

Ju-Seog Lee

USA

Soo-Chin Lee

Singapore

Georg Lenz

Germany

Javier Leon

Spain

Kiat Hon Lim

Singapore

Chen-Ching Lin

Taiwan

Jan Lindeman

Netherlands

Sara Lindstrom

USA

Da-Zhi Liu

USA

Ching-Ti Liu

USA

Nianjun Liu

USA

Holly Longstaff

Canada

Manuel Lopez-Perez

Spain

Jiachun Lu

China

Ingrid Lundberg

Sweden

Aldons Lusis

USA

Yves Lussier

USA

Anselm Mak

Singapore

Upender Manne

USA

Silva Markovic-Plese

USA

David Martin

USA

Gabor Matyas

Switzerland

Nadine Melhem

USA

Lourdes Mengual

Spain

Katherine Mitsouras

USA

Stefano Monti

USA

Gudrun Moore

UK

Carlos Moreno

USA

Patrice Morin

USA

David Morris

UK

Apiwat Mutirangura

Thailand

Samuel Myllykangas

Finland

Nimesh Patel

UK

Sven Nelander

Sweden

Alain Nepveu

Canada

Emily Noonan

USA

Michael Ochs

USA

Michael Oldham

USA

Bob Olsson

Sweden

Luz Orozco

USA

Joaquín Ortego

Spain

Csaba Ortutay

Finland

John Phan

USA

Kristian Pietras

Sweden

Vlad Popovici

Czech Republic

Robert Prins

USA

Milica Putnik

Sweden

Ignacio Rego-Pérez

Spain

Jorge Reis-Filho

UK

Luisella Righi

Italy

Matthew Ritchie

Australia

Christophe Rocher

France

Alexandra Rosa

Portugal

Eric Rouchka

USA

Jianhua Ruan

USA

Andrey Rzhetsky

USA

Toshiharu Sakurai

Japan

Paul Sandor

Canada

Ian Sayers

UK

Regien Schoemaker

Netherlands

Marco Scotti

Italy

David Serre

USA

Sunita Setlur

USA

Thomas Seyfried

USA

Kui Shen

USA

Anieta Sieuwerts

Netherlands

Xueling Sim

USA

Diane Simeone

USA

Richard Skipworth

UK

Marcin Skrzypski

Poland

Alicia Smith

USA

Maud Starmans

Canada

Justin Stebbing

UK

Jyothi Subramanian

India

Ruping Sun

Germany

Thomas Sutter

USA

Charles Swanton

UK

Jane Synnergren

Sweden

Maurizio Taglialatela

Italy

Zohreh Talebizadeh

USA

Ene-Choo Tan

Singapore

Anne-Marie Tassé

Canada

Elizabeth Thomas

USA

Kelsie Thu

Canada

Lisbeth Tranebjaerg

Denmark

Carl Troein

Sweden

Jennifer Troyer

USA

Ander Urruticoechea

Spain

Shin-Ichi Usami

Japan

Ignacio Varela

Spain

Marquis Vawter

USA

Vidya Velagapudi

Finland

Zoraida Verde

Spain

Sophie Verrier

Switzerland

Ida Vogel

Denmark

Benjamin Voight

USA

Volker Wacheck

Austria

Ryuichi Wada

Japan

Zuyi Wang

USA

Xingbin Wang

USA

Charles Warden

USA

Lucy Wedderburn

UK

Ashani Weeraratna

USA

Zhi Wei

USA

Peter White

USA

Thomas Wiegers

USA

Erik Wiemer

Netherlands

Pratyaksha Wirapati

Switzerland

Thian-Sze Wong

Hong Kong

Christopher Wong

Singapore

Zheyang Wu

USA

Albert Yang

Taiwan

Koichi Yoshimura

Japan

Jinsheng Yu

USA

Katherine Yutzey

USA

Tanja Zeller

Germany

Gangqiao Zhou

China

## Pre-publication history

The pre-publication history for this paper can be accessed here:

http://www.biomedcentral.com/1755-8794/6/6/prepub

